# Factors that influence modern contraceptive use among women aged 35 to 49 years and their male partners in Gomoa West District, Ghana: a qualitative study

**DOI:** 10.1186/s41182-023-00531-x

**Published:** 2023-08-03

**Authors:** Amy Takyi, Miho Sato, Michael Adjabeng, Chris Smith

**Affiliations:** 1 School of Tropical Medicine and Global Health, Nagasaki University Japan, 1-12-4 Sakamoto, Nagasaki, 852–8523 Japan; 2 World Health Organization (WHO) Country Office Accra, Korle-Bu, Box KB 493, Accra, Ghana; 3 Department of Clinical Research, London School of Hygiene and Tropical Medicine, Keppel Street, London, WC1E7HT UK

**Keywords:** Family planning, Contraception, Older women, Ghana

## Abstract

**Background:**

Fertility declines with age, but it remains important to protect women from unplanned pregnancies throughout their reproductive lives. The objective of this study was to describe factors that influence modern contraceptive use among women aged 35 to 49 years and their male partners in Gomoa West District of Ghana.

**Methods:**

In-depth interviews were conducted remotely for 22 women, 15 male partners of the women interviewed and seven family planning (FP) providers. In all, a total of 44 participants took part in the study. Seven refusals were recorded, four females and three males. Four focus group discussions were organized for 21 participants who took part in the in-depth interviews. Data collected were transcribed and coded after exporting to Nvivo12 qualitative analysis software. Thematic analysis was undertaken using an abductive approach.

**Results:**

Factors that influenced the use of modern contraceptives included: achieved desired family size, providing for the family, counselling by health professionals, influence of the male partner, and health reasons. Barriers cited included: religious or socio-cultural reasons, experience or fear of side effects, rumors or misconceptions, declining fertility, and the belief that contraceptive use is a matter for women. Within the study group, roughly half of women used modern contraceptives, while the majority of male partners were non-users.

**Conclusion:**

Contraception among women aged 35 to 49 years and their male partners is influenced by several factors such as achieved desired family size, influence of the male partner, rumors or misconceptions, and declining fertility. Strengthening male involvement in family planning activities and health educational activities could alleviate fear and reduce misconceptions about using modern contraceptives.

**Supplementary Information:**

The online version contains supplementary material available at 10.1186/s41182-023-00531-x.

## Background

Contraception enables individuals and families to manage fertility by reducing unintended pregnancy, abortions, pregnancy-related morbidity, and death (WHO, 2014). The use of contraception among women aged above 35 years has been reported to be low in low- and middle-income countries (LMICs) [1]. The low utilization of contraceptives and the use of less effective contraceptives methods have resulted in the occurrence of unplanned pregnancy among women in this age group in sub-Saharan Africa and was reported to be highest among women aged 40 to 44 years [2]. In 2014, among women currently married women in Ghana, the use of modern contraceptives was 26% for women aged 35 to 39 years, 28% for aged 40 to 44, and 22% for aged 45 to 49 years [3]. Additionally, the annual reports of the Gomoa West district for the 5-year period 2017 to 2021 showed that the number of abortions recorded among women aged 35 to 49 years was 183, with the year 2019 recording the highest with 52 abortions; which is higher than the 145 abortions recorded among adolescents for that period. Undesirable consequences of pregnancies among women above 35 years include preeclampsia, placenta abruption, and placenta previa; which increase significantly with age [3]. Moreover, advanced maternal age is a risk factor for spontaneous miscarriage; and is reported to be 50% among women aged 42 years and 75% among women aged 45 years. Moreover, advanced maternal age has negative effects on neonatal outcomes, such as small gestational age, fetal distress, and stillbirth [4].

The perception of men's role in family planning has shifted in recent times, acknowledging their active participation rather than just being supportive partners [5]. Factors such as educational level, socioeconomic status, fertility preferences, and the belief that contraception as a woman's responsibility are some predictors of modern contraceptive use among men. Engaging men in discussions about family planning with healthcare providers has shown to have an impact on their contraceptive use, as reported in other studies [6, 7].

In Ghana, there is a high level of knowledge about contraceptive use among men, being 97% across all age groups. However, the use of male condoms decreased from 2.4 in 2008 to 1.2 in the 2014 Ghana Demographic and Health Survey (2014). Despite changing views on family planning among men, some male partners still oppose the use of contraceptives by their female partners. The influence of husbands or male partners is a significant factor among both contraceptive users (62%) and non-users (63%), as noted by Staveteig (2016).

Contraception among women aged over 35 years is important due to the risk of unplanned pregnancy and poor obstetric outcomes. Even though the use of contraception increased globally from 42% in 1990 to 49% in 2019 [8], the contraceptive prevalence rate has been low in Sub–Saharan Africa, especially in Central and West Africa [8]. The type of contraception used varies according to the age of women [5]. It has been reported that in sub-Sharan African short-acting contraceptives are commonly used among women who want to limit childbearing, as well as older and married women; compared with long-acting reversible contraceptive (LARC) methods which have long duration of protection and low failure rate [6–10, 11]. The use of traditional contraceptive methods (TCMs) among older women could be due to a low perceived risk of pregnancy, as well as unstable relationships [9, 10].

Furthermore, the desire for more children, fear of side effects, and poor access to modern contraceptives could influence the use of traditional contraceptive methods [7]. Among married women, the influence of husbands was identified as an important factor in the use and nonuse of modern contraceptives among women in Ghana [10]. The peculiar characteristics of women in the 35 to 49 age group make it important to engage their male partners to understand the vulnerability and risks of their wives to poor maternal outcomes and the importance to use modern contraceptives to prevent unintended pregnancy. Moreover, the 2014 DHS survey and several conducted studies [2, 12] reported that even though the knowledge on contraception is over 95%, contraceptive use decreases with age and the proportion of unwanted pregnancy increased and was highest among women aged 45 to 49 years. While factors influencing contraceptive use among women aged 15 to 49 have been reported by several quantitative studies [11–22], there is a scarcity of information to understand the factors that influence contraception use among women aged 35 to 49 years and their male partners. This paper aims to examine the barriers and factors that promote modern contraceptive use among women 35 to 49 years and their male partners.

## Conceptual framework

**Fig. 1:**
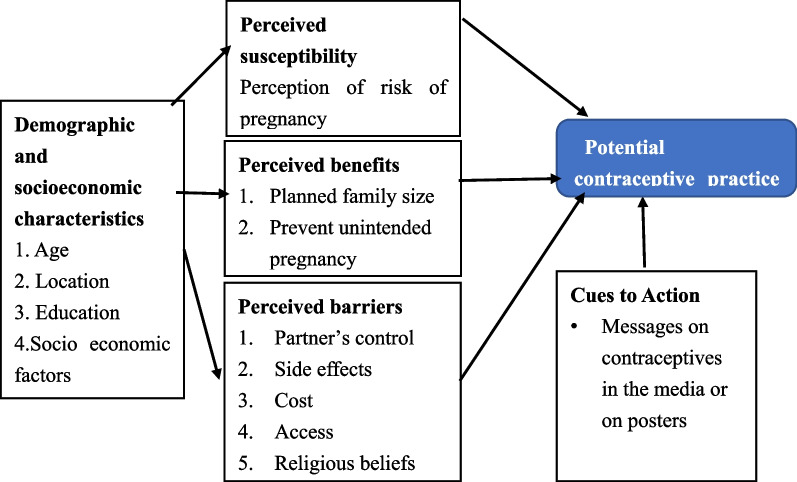
Conceptual framework on factors influencing modern contraceptive use. Reference: adapted from Hall [23]

This study adopted the conceptual framework of Hall (2012), with modifications bringing in the constructs of the health belief model to study factors that influence the use of modern contraception. The framework envisages that demographic and socioeconomic factors influence the likelihood of contraceptive use, including, perceived benefits, barriers, susceptibility, and clues to actions [23] (see Fig. 1).

## Methods

### Study design and setting

This was a qualitative study conducted using semi-structured in-depth interviews (IDIs) and Focus Group Discussions to understand and describe the experiences of women aged 35 to 49 years and their male partners about the use and nonuse of modern contraception. The study district was in the Central region of Ghana. Gomoa West has received support from USAID over the years to improve family planning coverage. However, the uptake of contraception among women aged 10 to above 35 years has recently fluctuated from 20 to 22% from 2018 to 2020, according to data obtained from the Ghana District Health Information Management software. Within the district, the Apam and Oguaa sub-districts were selected because they are the largest urban coastal and rural farming communities where participants with diverse socio-demographic and economic backgrounds could be purposefully identified.

The Ghana Statistical Service estimated the district population to be 135,189 according to the 2010 population census, with 45% males and 55% females. More than half (57%) of the population resided in rural areas. Most residents are of the Fanti ethnic group; however, there are small settlements of Ewes, Gas, and other ethnic groups along the coast who are engaged in the fishing industry.

### Sampling strategy

The study adopted the inclusion criteria developed by Westoff and Ochoa (1991) to guide the selection of participants [24]. That is, women aged 35 to 49 years who were currently married or in a sexual union, using or not using modern contraceptives, not pregnant, and had lived in the district for at least one year. Additionally, family planning service providers who worked in public health facilities within the District were included. The health staff had at least one year of working experience. The ages and use of contraceptives among family planning providers were not considered in the inclusion criteria. Male partners were recruited after permission was obtained from the women who had already participated in the interview. It was important to explore the perspective of men on contraception because of the influence they have on the uptake of contraceptives among women [10, 24–26]. A user is defined as a participant who used any of the modern contraceptive methods; specifically, male sterilization, oral contraceptives, implants, injectables, diaphragms, vaginal ring, intrauterine devices, male and female condoms, female sterilization, patches, lactational amenorrhea method, fertility awareness methods, cervical cap, spermicidal agents, or emergency oral contraception pills [WHO (2018)]. Non-users were participants who did not use any modern contraceptive method.

### Data collection procedures

Semi structured in-depth interviews (IDIs) were conducted remotely via WhatsApp [27] for 22 women aged 35 to 49 years; as well as 15 male partners of women and 7 family planning providers. The Principal Investigator with support from three trained on-site research assistants (one female, two males), facilitated the remote data collection from February 1st to 22nd, 2021. The research team explained procedure for the remote data collection to participants. After the participants have been taken through the information sheet and informed consent form to conduct the interview, a research assistant connected the Principal Investigator via the WhatsApp application. Participants were selected purposively to consider participants from urban and rural communities with diverse socio-cultural backgrounds who were users and non-users of modern contraceptive methods. Overall, 44 participants took part in the IDIs.

To observe a common opinion of informants about contraception in rural and urban communities, an additional 4 focus group discussions (FGDs) were organized remotely via Zoom [28] for women aged 35 to 49 years and their male partners who were users and nonusers. The IDI topic guide was developed separately for women aged 35 to 49 years, male partners, and family planning providers. The topic guide included the subjects of knowledge, perception and utilization of modern contraceptives, motivators and barriers to using modern contraceptive methods, perceptions of male partners about modern contraception, and the influence of a male partner on modern contraceptives use. The FGD topic guide had open ended prompts which covered topics on their experiences in using modern and traditional contraceptives, the reasons for nonuse, and the influence of the male partner on contraception.

The research assistants had tertiary education in community health and public nursing. They were trained virtually through Zoom for two days. On the first day the research assistants were informed on the research rationale, ethical considerations, and data collection tools. On the second day, the data collection tools were revised and modified to reflect the feedback received after pretesting in a neighboring district.

Participants who used modern contraceptive methods (MCMs) were selected from the family planning clinics of two health centers. Non-users of modern contraceptives were identified and contacted face to face through home visits by research assistants with community health nurses and community health volunteers. The male partners were recruited after permission was obtained from the women who had already participated in the interview. Using the staff list from the district, the research team worked with the seven sub-district heads to select the family planning providers who were public health nurses, general nurses, and community health nurses. Twenty-one IDI informants with diverse backgrounds who were interviewed during the IDIs also consented to participate in four FGDs facilitated by the principal investigator (PI) with the support of three research assistants.

The topic guides for IDIs and FGDs were developed separately for users, non-users, male partners, and family planning providers. The IDIs and FGDs were conducted in English, or the local Fante languages if preferred by the participant, and audio was recorded with permission. The IDIs were conducted via WhatsApp on a smartphone. The participants were interviewed at their homes during the IDIs. The FGDs were held on the school compound and health facility. The IDIs and FGDs were organized from February 1st to 22nd, 2021. Eighteen participants were contacted twice to obtain more information.

Key ethical issues were explained to all participants prior to data collection. Evidence of their voluntary participation was signified by signing or thumb printing an informed consent form. The procedure for the remote data collection was explained. Participants who preferred to use their mobile phones for the remote interview were given a ten Ghana cedis (GH 10.00) [US$ 1.68] recharge card. Participants who did not have the WhatsApp application on their mobile phone, or those who preferred to use the mobile phone purchased for the research assistants for data collection, were allowed to use the mobile phone. After the participant was taken through the information sheet and informed consent form, a research assistant connected the principal investigator via the WhatsApp application.

Due to the remote nature of the study, research assistants were trained to keep field notes to capture nonverbal communication and other observations. The principal investigator also kept fieldnotes and discussed them with the research assistants after each interview. The principal investigator asked further probing questions after reflecting on the response from the participants to clarify any ambiguity and ensure accuracy and consistency in data collection. The average duration of interviews among women and their husbands was 55 min and 58 min among family planning providers. The FGDs lasted for an average duration of 96 min.

Data saturation was reached between 39 to 44 participants. The principal investigator worked at Gomoa West District Health Directorate and is familiar with the study area. To reduce bias, the principal investigator examined her values, assumptions, and prior understanding brought into the study. The principal investigator also kept a field note to document her thoughts and observations during the research process [11].

### Data analysis

Data were transcribed from the local Fante language to English. Data analysis was flexible, iterative, and simultaneously conducted during data collection. A thematic analysis by Braun and Clarke (2006) was adopted. to identify, analyze, and report patterns within the data collected [27]. Coding was done on two levels. First, inductive coding was done by using descriptive coding. This approach gives a complete picture of the themes which emerged from the data. Secondly, to answer the research questions, a deductive coding was subsequently performed using research objectives. The first author transcribed the data and read the interview transcripts and field notes several times, to be conversant with the data collected and to identify the main themes. Three authors reviewed the identified themes. Data were managed using Nvivo12 qualitative analysis software.

The patterns of similarities and differences were identified from the codes generated; which were put into sub-categories, categories, and themes. The themes identified were examined, renamed, and merged to reflect the research objectives. The codes and categories under each theme were examined to ensure that they were descriptive of the themes. Participants were assured of privacy and confidentiality of the information they provided. Pseudonyms were used to store participant information. All participants’ responses reported during the findings of the study were disguised to ensure anonymity without distorting the data content. All soft copies of participant information, interview transcripts, field notes, and audio recordings were stored on the principal investigator’s personal computer; which was protected by a password, backed up on a password protected external hard drive, and kept in a secure locker. Some quotations of participants were extracted as evidence of response. Ethical approval was obtained from Nagasaki University (Ref: NU_TMGH_2020-146–1) and Ghana Health Service Ethics Review Committees (Ref: GHS-ECR028/11/20). The Standards for Reporting Qualitative Research (SRQR) checklist is available as an Additional File 1.

## Results

The socio-demographic characteristics of the participants are summarized in Table 1. Seven people refused to participate in the study; and four women (two users and two non-users) did not permit the research team to interview their husbands. The reasons given were that the husbands were not aware that the participants used modern contraceptive methods, others presumed that their husbands would not be interested in the interview, and some were unwilling to give out phone numbers of their male partners who had travelled. It was revealed during IDIs that all non-users of modern contraceptive methods used traditional contraception (such as periodic abstinence method and withdrawal), which was not considered a form of contraception by the participants. In the study community, modern contraception was synonymous to contraception. The median age of women was 38.5 years. All 15 male partners of the women interviewed were older than their female partners. Christianity was the most common religion among all participants. Most (57%) of the family planning providers were community health nurses with a median age of 31 years. Injectables were the most commonly used modern contraceptive methods, whereas periodic abstinence was prevalent among non-users of modern contraceptive (MC). Five male partners had ever used a male condom, and two were current users. Among women, 55% used modern contraceptive, 23% had never used any modern contraceptive method, and 18% were former modern contraceptive users. Roughly 4% used both modern contraceptives and traditional contraceptive (TC) methods. Among male partners, a majority (67%) reported never having used a male contraceptive; and 33% had ever used male condoms before, broken down to 20% were previous users and 13% were current users. Two major themes were identified: facilitators of modern contraceptive use and barriers of modern contraceptive use.

**Table 1:** Characteristics of participants: women aged 35–49 years and their male partners

Characteristics	Number (N)
Median age of participants (years) N = 44	
Female	41
Male	47
Education (female participants only) N = 22	
None	2
Primary	5
Secondary	8
Tertiary	7
Type of contraception currently using N = 22^a^	
Depo provera	15
Norigynon	3
Implanon	1
IUD	1
Jadelle	2
Pill	6
Achieved ideal family size (by female N = 22	
Yes	14
No	7
Not sure	1
Type of contraceptive ever used (by non-users) N = 11	
Depo provera	5
Norigynon	0
Implanon	0
IUD	0
Jadelle	1
Pill	0
Intention to discontinue the use of MCM in the future (by users of MCM) N = 11	
Yes	0
No	2
Other (If I experience serious side effects)	9
Woman gave permission to interview male partner N = 22	
Yes	18
No	4
Ever used male condoms (by male participants) N = 15	
Yes	2
No	13
Intention to use male contraception (by male participants) N = 15	
Yes	0
No	`13
Maybe	2

*IUD* Intrauterine device, *MCM* Modern Contraceptive Method

^a^Frequency of modern contraceptive methods by women

### Factors that promote the use of contraceptives

This theme describes the factors that promoted contraception, such as achieved desired family size, providing for the family, counselling by health staff, influence of the male partner, and health reasons. As indicated in the adapted health belief model, these categories can be put under perceived susceptibility, perceived benefit, and cues to action.

#### Perceived susceptibility

##### Achieved desired family size

Seven participants who used modern contraceptive methods indicated that they had achieved the desired family size.

“After given birth to four children, I decided to do the five years family planning [Jadelle®], so when I got to the clinic, I told them what I wanted to do.” (IDI, MCU 3).

Two women who were nonusers expressed their intention to use modern contraceptive methods after giving birth to the number of children they desired.

“When I give birth to the 5 children I desire, then I will do family planning to prevent any unplanned pregnancy.” (1D1, NMCU 2).

There were conflicting views on the achievement of the desired family size. Among seven couples who used modern contraceptives, four husbands indicated that, they had achieved the desired family size; however, their wives wanted to have one or two more children. The majority of women who were non-users of modern contraceptives desired more children (6/12), although most (9/11) of their husbands had achieved the desired family size; and four of the male partners had sometimes used a male condom during unsafe periods of their menstrual cycle.

#### Perceived benefit

##### Providing for the family

Four informants mentioned that controlling the family size using contraceptives enables them to provide for the family.

“Contraceptive helps me to space my children and also save some money because I have time to work.” (FGD, H-MCU 4).

##### Health reasons

Some participants mentioned that pregnancy at an advanced maternal age was dangerous. A couple agreed on female sterilization after their 5^th^ child because of a perceived risk to poor maternal outcomes in subsequent pregnancies.

“When my wife gave birth to the 5th child, she bled profusely. I was worried about her life and health if she gets pregnant again. So, we agreed and the doctors did the sterilization for her.” (IDI, H-MCU 6).

##### Cues to action

*Counselling by health staff* Five participants mentioned that counselling and health education by health workers encouraged them to use modern contraceptive methods. A mother of five children stated regarding her experience on the advice of health workers to use modern contraceptive methods:

“For me, if they [the doctors] had not told me to come and do it, I wouldn’t have done family planning.” (IDI, MCU 5).

Most non-users (10/11) did not receive counselling on effective ways to avoid pregnancy without using modern contraceptives. Conversely, family planning providers mentioned conducting health education on traditional contraceptive methods.

“A lot of them too are concerned with the side effects so we counsel them on the traditional methods, the advantages and disadvantages.” (IDI, FPP 5).

##### Influence of the male partner

Husbands played a significant role in choosing and using modern contraceptive methods. Eight male partners confirmed this. After making a joint decision to plan their family, a husband of a participant accompanied her to buy the pills at a drug store, as she was shy because it was her first time to use a modern contraceptive method. Among non-users, two participants were discouraged by their husbands from using modern contraceptives because of the fear of side effects.

“I will not recommend women to use the modern contraceptives. During their unsafe period, their husbands can abstain from sex or use the male condom.” (IDI, H-NMCU 4).

Health staff conducted various integrated activities to engage men on family planning.

“We usually organize community meetings and health education in churches and at the health center. Recently, we held a stakeholder meeting on family planning and invited all the opinion leaders for a meeting on health issues including family planning” (IDI, FPP 1).

### Barriers to contraceptives use

This theme describes factors that were perceived to hinder modern contraceptive use, such as religion or socio-cultural reasons, experience or fear of side effects, declining fertility, rumor or misconceptions, declining fertility, and the belief that contraceptive use is a matter for women, physical access and cost.

#### Religion reasons

All seven of the family planning providers and eight participants identified this theme as one of the reasons for nonuse of modern contraceptive methods. The family planning providers stated that the activities of Pastors discouraged women from using modern contraceptive methods.

“There is a Pastor in Kyiren community, who tells his congregation not to do family planning, so most of the women there do not do it. Others hide and come for family planning.” (IDI, FPP 3).

Six informants reported that the use of contraceptives is a sin.

“They think that it is a sin to use anything to prevent pregnancy. They prefer natural method.” (FGD, H-NMCU 8).

#### Socio-cultural reasons

Socio-cultural factors were prominent among men as reasons for the nonuse of male condoms. Eight male partners mentioned that using a male condom was a common practice among unmarried young men who engaged in premarital sex. Married men who used male condoms were seen as promiscuous. This perception may have influenced the low utilization of male condoms among married men.

“Most of the community members think that a married man should not use male condom, it is the young men who are not married who should use it. When they see a married man going to buy it, they think that he is cheating on his wife.” (IDI, H-NMCU 5).

However, four male partners would use a male condom if their wives recommended it.

#### Experience or fear of side effects

The experience and fear of side effects prevented five women and two husbands from using MCMs. Seven informants had experienced some side effects. Most (5/7) of these informants used Depo Provera.

“Some of us have used family planning before but we have to stop because of the side effects.” (FGD, NMCU 8).

“Some of the women do not do family planning but they are afraid of the side effects. If the manufactures can do sometimes about the side effects it will be very good.” (IDI, FPP 4).

Five participants had never used modern contraceptive methods because of the side effects their friends and relatives had experienced. Two male participants who used male condoms discontinued because their wife had a rash on her vulva, and another disliked the use of male condoms. Additionally, a 41-year-old husband stopped using male condoms due to latex allergy.

#### Perceived male partner disapproval of male contraceptives

The new theme that emerged during data analysis. Four women assumed that their male partners disliked the male condom, whereas their male partners stated that they would use it if their wives recommended it. Six male participants stated that most husbands found it difficult to bring up the topic of male contraceptives in marriage because they felt uncomfortable discussing it. There was inadequate communication among couples on the use of male contraception due to cultural beliefs, even though some male partners preferred to use male condoms rather than having their wives using modern contraceptives. Due to cultural sensitivity around male contraception, male partners recommended family planning providers to encourage women to attend the clinic with their husbands to discuss such issues.

#### Rumor or misconceptions

Five out of seven family planning providers indicated that rumors or misconceptions were primary concerns that prevented the target group from accessing modern contraceptive methods.

“I also heard that when women do family planning, they commodities can get lost in the body and this can lodge in the heart and cause hypertension.” (FGD, H-MCU 8).

#### Declining fertility

Three informants reported that, in the community, the nonuse of modern contraceptives among women in the target group is the perception of declining fertility as women transition to menopause.

*“Sometimes, when women are getting older, they think that they don’t have to use contraceptives. So, I think that age is a very important factor.* (FGD NMCU 10)”

#### The belief that contraceptive use is a matter for women

Four male partners indicated that they considered family planning a woman’s issue and did not encourage men to use male condoms. All the family planning providers mentioned that most men do not participate in family planning activities. Consequently, health staff found innovative ways to attract men to these activities.

“If the men know that the topic is on family planning, they will not come. Now, when we organize activities, we do not tell them it is on family planning. We inform them that we are coming to talk to them about health issues, that way, the men will come. We add checking of blood pressure, and body mass index to our health educational programs to attract men too.” (IDI, FPP 3).

Not all men disapproved of the use of male condoms. For example, two male partners encouraged men to use male condoms because they considered them effective and without side effects. The reported side effects of modern contraceptives were headaches; dizziness; heart palpitations; and irregular, ceased, or excessive menstruation. Almost all the modern contraceptive methods users were informed of potential side effects.

“When I hear of the side effects of contraceptives, I think men should be encouraged to use the male condom to protect their wives from unplanned pregnancy.” (IDI, H-NMCU 10).

#### Physical access

Physical access to contraceptives was identified only in rural communities. A 45 year—old woman with 3 children stated that distance and transportation issues to the health facility, situated on the outskirt of the community, prevented some women from accessing modern contraceptives. Access to the facility by foot takes about 30 min and costs GH₵ 5.00 [US$ 1.11] by taxi.

One family planning provider working in a rural area reported:

“…It’s a bit far. The women who do not want to come to the health center leave their FP cards with me. So, when the date is due, I check and take their FP commodities with me when I am going home. Not me alone but all the staff at the family planning unit. We all have clients we have this arrangement with, so we do that for them.” [IDI, FPP 2].

#### Cost

The cost of contraceptives was said to be affordable among most participants, and only one participant mentioned that she could not afford long-term modern contraception, which she desired to use. She had no formal education, with six children, lived in a rural community, and depended on her husband to support her financially to get access to the contraceptive.

“I don’t have money. If I have some money, I would have gone to the clinic myself to do the family planning and not wait for my husband to come before I go.” [IDI, H-NMCU 9].

Two family planning providers indicated that they sometimes provided family planning service on credit to clients who could not afford modern contraceptives.

## Discussion

### Summary of main results

This study reported that the use of modern contraceptives among women 35 to 49 years and their male partners was motivated by various factors; including an achieved desired family size, providing for the family, counselling by health professionals, influence of the male partner, and health reasons. The barriers to modern contraceptive use included religious and socio-cultural reasons, experience or fear of side effects, perceived male partner disapproval of male contraceptives, rumors or misconceptions, declining fertility, and that contraceptive use is a matter for women rather than men. Other barriers were physical access and cost.

### Interpretation and comparison with existing literature

The factors that influence the use of modern contraceptives are similar for women aged 15 to 49, as reviewed in the literature [21–25]. However, other findings are distinct to women aged 35 to 49 years and male partners; such as the influence of the male partner, health reasons, declining fertility, and the belief that contraceptive use is a matter for women rather than men [24–26].

The findings that achieved desired family size, counselling by health professionals, health reasons, and the influence of the male partner promoted contraceptive use among women 35 to 49 years and their male partners are consistent with what has been found by studies among couples and married women in the 15 to 49 years age group in Ghana [11], Nigeria [3, 14], and Kenya [15].

Health professionals provide information and counseling that promote using contraceptives. The activities of health professional created opportunities to engage male partners, address health concerns, correct misconceptions, and provide accurate information about modern contraceptive methods. This finding is corroborated by a study conducted in Ghana [11, 17, 18] and in sub-Saharan Africa [19–24].

Religious beliefs have been explored as one of the determinants of modern contraceptive use. In this study, religious beliefs were identified as a major barrier to contraception among married men more than married women. Among married men, the use of male condoms was perceived by some as a sin, and against their religious beliefs. However, among married women who were Catholics and Muslims, religious beliefs did not prevent them from using modern contraceptive methods, even though family planning providers stated that some religious leaders in the communities discouraged women from using modern contraceptive methods. These participants explained that it was against their faith to have a large family and not be able to provide for them. This result resonates with a study conducted in Ghana [10], which reported both positive and negative influences of religion on contraceptive use. The negative influence of religion is in line with studies from research in Ghana [25], Ethiopia [26], and Nigeria [29].

A major concern of using modern contraceptive methods (MCMs) is possible side effects, as has been established by several studies conducted in LMICs and sub-Saharan Africa [11, 14, 27, 30]. The experience of side effects resulted in discontinuation of MCMs, switching between MCMs, or shifting to traditional contraceptive methods. Moreover, the fear of side effects as a result of the experiences of friends or close relatives was a reason for the nonuse of MCMs, as reported by studies in Ghana [10, 28–33]. The roles of rumor or misconceptions are described in the literature as a typical barrier to using modern contraceptives. Findings from this study showed that, among married women, that myths and misconceptions about MCMs influenced their attitude and use of traditional contraceptive methods. The sources of misconceptions stem from herbal drug peddlers, previous users of modern contraceptive methods, and community members. Currently, the use of herbal medicines in Ghana has gained popularity, reaching people through television, radio advertisements, and word of mouth. Dali et al. reported that in Ghana, some herbal medicines are used as contraceptives and to cause abortion [34, 35]. As indicated by Kleinman’s explanatory model [36], popular and folk sectors co-exist in society as alternatives to the professional health sector for providing health care [37]. Improvements in the level of education among women could promote better understanding of modern contraceptive methods and reduce misconceptions towards modern contraceptive methods [38].

Perceptions of their partner’s approval of a specific contraceptive method were a predictor of contraceptive use among men [39]. Among married men, consent from their wives enabled them to use male condoms to prevent unplanned pregnancies, particularly among non-users of modern contraceptives. Building consensus on the use of male contraceptives is essential because of the perception that married men who use male condoms are promiscuous [40, 41].

Declining fertility among women as they approach menopause can be interpreted as lowering the risk of pregnancy, and has been reported by several studies as a reason for modern contraceptive nonuse [9]. Male partners view contraceptives as an issue that is for females to solely consider. Most married men support their wives’ use of modern contraceptive methods. However, due to the experience or fear of side effects and health concerns, some husbands use male condoms to protect their wives from unintended pregnancy, especially among couples who use traditional contraceptive methods. Moreover, men view that family planning programs target women only; hence their involvement is low. Other studies also reflect such male perspectives of family planning programs [17, 36, 42–45]. Over the years, access to health facilities in Ghana has improved through the Community-Based Health Planning and Services (CHPS). However, in some communities, the location of health facilities is situated far from inhabitants [1]. Within communities, health workers provide family planning services to women through regular home visits. Although it was not a major barrier, the distance to a health facility was a key challenge in one sub-district, preventing people from accessing the health facility for health, including family planning services. This finding is consistent with previous studies carried out in LIMCs [2, 16, 46, 47]. In Ghana, Eliason and colleagues (2014) reported that women who lived within a 5 km radius of the nearest health facility are more likely to use contraceptives [3].

The cost of LARCs was not a major determinant for contraceptive use among most couples. However, women with lower educational levels, unemployed status, and who lived in more impoverished families considered the cost of LARCs to be high. In Ghana, the cost of LARCs is relatively higher than short-acting reversible contraceptives [48–50]. This finding is consistent with studies conducted in sub-Saharan Africa [4, 8, 51, 52] and in Ghana [5, 6]. This study revealed a varied perspective among male partners on modern contraception. However, most male partners expressed a preference for modern contraceptives as an alternative to exposing their wives to the potential adverse effects of modern female contraceptives.

These findings highlight the importance of health workers devising strategies to actively involve male partners in discussions about contraception, as they have a significant impact on the utilization of modern contraceptives. When adequately engaged in family planning programs, male partners can not only influence contraceptive use but also become potential users themselves. Moreover, the health promotion unit of the Ghana Health Service could create informative audio messages on modern contraceptives, which can be broadcasted through community FM stations or disseminated via information centers. This initiative aims to combat the dissemination of misinformation by certain individuals involved in the illegal sale of drugs, who may spread inaccurate information about modern contraception. Furthermore, the inclusion of family planning into the National Health Insurance Scheme in 2022 by the Government of Ghana is commendable. Sustained promotion of this information has the potential to further increase the uptake of modern contraceptives.

## Strengths and limitations

A strength of this study is the triangulation of information by interviewing the male partners of women and family planning providers. This provided the opportunity to obtain the perspectives of male partners on contraception, their influence on the use and nonuse of modern contraceptive methods among women, which is the unique strength of this study. Additionally, FGDs were conducted to observe a common view of informants about contraception.

This study has several limitations. The qualitative methodology used limits generalization of the findings. The remote nature of the study reduced the opportunity for the PI to continuously observe nonverbal communications by participants. The participation of male partners was at the discretion of the women interviewed. While this approach allowed us to triangulate the information, it limited male partner involvement in the study. Future quantitative research could investigate barriers that hinder couples from accessing and utilizing modern contraceptive methods. By exploring these barriers, researchers can provide valuable insights into the factors contributing to low uptake of modern contraceptives among couples. This knowledge can then be used to develop effective family planning programs and improved reproductive health outcomes.

## Conclusions

Contraception among women aged 35 to 49 years and their male partners is influenced by several factors. Women used some form of contraceptive due to a heightened perception of their susceptibility to pregnancy. Women who used traditional contraceptives did not consider traditional contraceptive methods as a form of contraception.

We propose that intensifying health education, and engaging women and their male partners could improve the use of contraceptives among women aged 35 to 49 years and their male partners.

### Supplementary Information

**Additional file 1. **Standard for reporting qualitative research (SRQR) checklist. Standard for reporting qualitative research (SRQR) checklist. The checklist for reporting qualitative research.

## Data Availability

The transcripts from which this manuscript was developed are available and can be obtained on request from the corresponding author.

## Abbreviations

EOC: Oral emergency contraception
FGD: Focus group discussion
FP: Family planning
FPP: Family planning provider
H**-**MCU: Husband of modern contraceptive user
H-NMCU: Husband non-modern contraceptive user
LMICs: Low- and middle-income countries
MCM: Modern contraceptive method
MCU: Modern contraceptive user
NMCU: Non-modern contraceptive user
PI: Principal investigator
TCM: Traditional contraceptive method
USAID: United States Agency for International Development

## References

[1] Vu LTH, Oh J, Bui QTT, Le ATK (2016). Use of modern contraceptives among married women in Vietnam: a multilevel analysis using the Multiple Indicator Cluster Survey (2011) and the Vietnam Population and Housing Census (2009). Glob Health Action.

[2] Ameyaw EK, Budu E, Sambah F, Baatiema L, Appiah F, Seidu AA (2019). Prevalence and determinants of unintended pregnancy in sub-Saharan Africa: a multi-country analysis of demographic and health surveys. PLoS ONE.

[3] Keogh SC, Otupiri E, Castillo PW, Chiu DW, Polis CB, Nakua EK (2021). Hormonal contraceptive use in Ghana: the role of method attributes and side effects in method choice and continuation. Contraception.

[4] Blomberg M, Tyrberg RB, Kjølhede P (2014). Impact of maternal age on obstetric and neonatal outcome with emphasis on primiparous adolescents and older women : a Swedish Medical Birth Register Study. BMJ Open.

[5] Hardee K, Croce-Galis M, Gay J. Men as Contraceptive users: programs, outcomes and recommendations, working paper. 2016. p. 1–69.

[6] Ali A, Zar A, Wadood A (2022). Factors associated with modern contraceptive use among men in Pakistan: evidence from Pakistan demographic and health survey 2017–18. PLoS ONE.

[7] Kabagenyi A, Ndugga P, Wandera SO, Kwagala B (2014). Modern contraceptive use among sexually active men in Uganda: does discussion with a health worker matter?. BMC Public Health.

[8] Cohain JS, Buxbaum RE, Mankuta D (2017). Spontaneous first trimester miscarriage rates per woman among parous women with 1 or more pregnancies of 24 weeks or more. BMC Pregnancy Childbirth.

[9] United Nations. World Fertility and family planning 2020 Ten key messages. Dep Econ Soc Aff. 2020, p. 2019–20. Available at: https://www.un.org/en/development/desa/population/publications/pdf/family/Ten_key_messages-for-WFFP2020_highlights.pdf. Accessed 12 June 2022.

[10] MacQuarrie KLD, Juan C, Allen C, Zweimueller S, Gemmill A. Women’s contraceptive profiles throughout the life course in Burundi and Nepal. DHS Anal Stud. 2018;(72):xiii-pp. Available at: https://dhsprogram.com/pubs/pdf/AS72/AS72.pdf. Accessed 10 Aug 2022.

[11] Boadu I (2022). Coverage and determinants of modern contraceptive use in sub-Saharan Africa: further analysis of demographic and health surveys. Reprod Health.

[12] Maggwa BN, Office AR. F am ily Health. 2008;2(1).

[13] Negash WD, Belachew TB, Asmamaw DB (2022). Long acting reversible contraceptive utilization and its associated factors among modern contraceptive users in high fertility sub-Saharan Africa countries: a multi-level analysis of recent demographic and health surveys. Arch Public Heal.

[14] Godfrey EM, Chin NP, Fielding SL, Fiscella K, Dozier A (2011). Contraceptive methods and use by women aged 35 and over: A qualitative study of perspectives. BMC Womens Health.

[15] Leeman-markowski BA, Meador KJ, Moo LR, Cole AJ, Hoch DB, Garcia E, et al. US. Department of Veterans Affairs. 2020;51(4):315–24.

[16] Rossier C, Corker J (2017). Contemporary use of traditional contraception in sub-Saharan Africa. Popul Dev Rev.

[17] Staveteig S. Understanding unmet need in Ghana: results from a follow-up study to the 2014 Ghana demographic and health survey. DHS Qual Res Stud No 20. 2016. http://dhsprogram.com/pubs/pdf/QRS20/QRS20.pdf.

[18] Ghana Statistical Service - GSS, Ghana Health Service - GHS, and ICF International. 2015. Ghana Demographic and Health Survey 2014. Rockville: GSS, GHS and II. 2014.

[19] Westoff CF, Ochoa LH. Comparative studies 5: unmet need and the demand for family planning. g. Institute for Resource Development; Columbia. 1991. Available at: https://dhsprogram.com/pubs/pdf/CS5/CS5.pdf. Accessed 6 May 2022.

[20] Manji K, Hanefeld J, Vearey J, Walls H, De Gruchy T (2021). Using WhatsApp messenger for health systems research: a scoping review of available literature. Health Policy Plan.

[21] Archibald MM, Ambagtsheer RC, Casey MG, Lawless M (2019). Using zoom videoconferencing for qualitative data collection: perceptions and experiences of researchers and participants. Int J Qual Methods.

[22] Preissle J. Subjectivity statement. The sage encyclopedia of qualitative research methods. 2008. p. 289–92.

[23] Hall KS (2012). The health belief model can guide modern contraceptive behavior research and practice. J Midwifery Women’s Health.

[24] Braun V, Clarke V (2006). Qualitative research in psychology using thematic analysis in psychology using thematic analysis in psychology. Qual Res Psychol.

[25] Bawah AA, Asuming P, Achana SF, Kanmiki EW, Awoonor-Williams JK, Phillips JF (2019). Contraceptive use intentions and unmet need for family planning among reproductive-aged women in the Upper East Region of Ghana. Reprod Health.

[26] Nonvignon J, Novignon J (2014). Trend and determinants of contraceptive use among women of reproductive age in Ghana. Popul Afr Stud.

[27] Wuni C, Turpin CA, Dassah ET (2017). Determinants of contraceptive use and future contraceptive intentions of women attending child welfare clinics in urban Ghana. BMC Public Health.

[28] Ajayi AI, Adeniyi OV, Akpan W (2018). Use of traditional and modern contraceptives among childbearing women: findings from a mixed methods study in two southwestern Nigerian states. BMC Public Health.

[29] Godfrey EM, Chin NP, Fielding SL, Fiscella K, Dozier A (2011). Contraceptive methods and use by women aged 35 and over: a qualitative study of perspectives. BMC Womens Health.

[30] Rominski SD, Morhe ES, Maya E, Manu A, Dalton VK (2017). Comparing women’s contraceptive preferences with their choices in 5 urban family planning clinics in Ghana. Glob Heal Sci Pract..

[31] Irani L, Speizer IS, Fotso JC (2014). Relationship characteristics and contraceptive use among couples in urban Kenya. Int Perspect Sex Reprod Health.

[32] Adongo PB, Tapsoba P, Phillips JF, Tabong PTN, Stone A, Kuffour E (2013). The role of community-based health planning and services strategy in involving males in the provision of family planning services: a qualitative study in Southern Ghana. Reprod Health.

[33] Eliason S, Awoonor-Williams JK, Eliason C, Novignon J, Nonvignon J, Aikins M (2014). Determinants of modern family planning use among women of reproductive age in the Nkwanta district of Ghana: a case-control study. Reprod Health.

[34] Zimmerman LA, Bell SO, Li Q, Morzenti A, Anglewicz P, Tsui AO (2019). Individual, community and service environment factors associated with modern contraceptive use in five Sub-Saharan African countries: a multilevel, multinomial analysis using geographically linked data from PMA2020. PLoS ONE.

[35] Dali GLA, Pappoe ANM, Akotoye HK (2019). Plants used as abortifacients and contraceptives in some communities on the fringes of subri river forest reserve in Ghana. Afr J Reprod Health.

[36] Kleinman A, Eisenberg L, Good B (2006). Culture, illness, and care: clinical lessons from anthropologic and cross-cultural research. Focus (Madison).

[37] Guure C, Maya ET, Dery S, Da-Costa Vrom B, Alotaibi RM, Rezk HR (2019). Factors influencing unmet need for family planning among Ghanaian married/union women: a multinomial mixed effects logistic regression modelling approach. Arch Public Heal.

[38] Solanke BL, Banjo OO, Oyinloye BO, Asa SS (2018). Maternal grand multiparity and intention to use modern contraceptives in Nigeria. BMC Public Health.

[39] Huber-Krum S, Norris AH (2020). Gender differences in perceived benefits of and barriers to use of modern contraceptive methods in rural malawi. Int Perspect Sex Reprod Health.

[40] Grabert BK, Speizer IS, Domino ME, Frerichs L, Corneli A, Fried BJ (2021). Couple communication and contraception use in urban Senegal. SAGE Open Med.

[41] Babazadeh S, Anglewicz P, Wisniewsk JM, Kayemb PK, Hernandez J, Bertran JT (2020). The influence of health facility-level access measures on modern contraceptive use in Kinshasa, DRC. PLoS ONE.

[42] Kassa M, Abajobir AA, Gedefaw M (2014). Level of male involvement and associated factors in family planning services utilization among married men in Debremarkos town, Northwest Ethiopia. BMC Int Health Hum Rights.

[43] Asa SS, Titilayo A, Kupoluyi JA (2018). Assessment of contraceptive use by marriage type among sexually active men in Nigeria. Int Q Community Health Educ.

[44] Tilahun T, Coene G, Luchters S, Kassahun W, Leye E, Temmerman M (2013). Family planning knowledge, attitude and practice among married couples in Jimma zone, Ethiopia. PLoS ONE.

[45] Kabagenyi A, Jennings L, Reid A, Nalwadda G, Ntozi J, Atuyambe L (2014). Barriers to male involvement in contraceptive uptake and reproductive health services: a qualitative study of men and women’s perceptions in two rural districts in Uganda. Reprod Health.

[46] Assefa L, Shasho Z, Kasaye HK, Tesa E, Turi E, Fekadu G (2021). Men’s involvement in family planning service utilization among married men in Kondala district, western Ethiopia: a community-based comparative cross-sectional study. Contracept Reprod Med.

[47] Asante FA. Cost of family planning services in Ghana. 2013. p 46. Available at: https://www.healthpolicyproject.com/pubs/243_GhanaFPCostingStudyFINAL.pdf. Accessed 7 Mar 2023.

[48] Agbenyo F, Marshall Nunbogu A, Dongzagla A (2017). Accessibility mapping of health facilities in rural Ghana. J Transp Health.

[49] Moreira LR, Ewerling F, Barros AJD, Silveira MF (2019). Reasons for nonuse of contraceptive methods by women with demand for contraception not satisfied: an assessment of low and middle-income countries using demographic and health surveys. Reprod Health.

[50] Amissah J, Nakua EK, Badu E, Amissah AB, Lariba L (2020). In search of universal health coverage: the hidden cost of family planning to women in Ghana. BMC Res Notes.

[51] Adedini SA, Omisakin OA, Somefun OD (2019). Trends, patterns and determinants of long-acting reversible methods of contraception among women in sub-Saharan Africa. PLoS ONE.

[52] Muanda M, Ndongo PG, Taub LD, Bertrand JT (2016). Barriers to modern contraceptive use in Kinshasa. DRC PLoS ONE.

