# Supplementary material for: Factors that influence modern contraceptive use among women aged 35 to 49 years and their male partners in Gomoa West District, Ghana: a qualitative study
Source: Trop Med Health. 2023 Aug 3;51:40. doi: 10.1186/s41182-023-00531-x (PMC10398952; doi:10.1186/s41182-023-00531-x)
Supplement: Supplementary file 1 — Additional file 1. Standard for reporting qualitative research (SRQR) checklist. Standard for reporting qualitative research (SRQR) checklist. The checklist for reporting qualitative research. [file 41182_2023_531_MOESM1_ESM.docx]

## **Authors**

Amy Takyi^1^ `

Miho Sato^1^

Michael Adjabeng^2^

Chris Smith^1,3^

## **Article title**

Factors that influence modern contraceptive use among women aged 35 to 49 years and their male partners in Gomoa West District, Ghana: a qualitative study.

## **Affiliations**

^1^ School of Tropical Medicine and Global Health. Nagasaki University Japan

1-12-4 Sakamoto, Nagasaki City 852 – 8523.

^2^ World Health Organization (WHO) Country Office Accra, Ghana

Box KB 493, Korle-Bu, Accra.

^3^ Department of Clinical Research, London School of Hygiene and Tropical Medicine, Keppel Street, London WC1E7HT, United Kingdom

## **Corresponding author**

Chris Smith

Email: [christopher.smith@lshtm.ac.uk](mailto:christopher.smith@lshtm.ac.uk)

Tel: +81(0)80-8710-5309

School of Tropical Medicine and Global Health, Nagasaki University, Sakamoto 1-12-4, Nagasaki 852-8523, Japan

|  | **Standards for Reporting Qualitative Research (SRQR)*** |  |
| --- | --- | --- |
|  | <http://www.equator-network.org/reporting-guidelines/srqr/> |  |
|  |  | **Page/line no(s).** |
| **Title and abstract** | |  |
|  | **Title** - Concise description of the nature and topic of the study Identifying the study as qualitative or indicating the approach (e.g., ethnography, grounded theory) or data collection methods (e.g., interview, focus group) is recommended | P1, lines 9-11 |
|  | **Abstract** - Summary of key elements of the study using the abstract format of the intended publication; typically includes background, purpose, methods, results, and conclusions | P2, lines 36-67 |
|  |  |  |
| **Introduction** | |  |
|  | **Problem formulation** - Description and significance of the problem/phenomenon studied; review of relevant theory and empirical work; problem statement | P4, lines 78-93  P7 lines 108 - 134 |
|  | **Purpose or research questio**n - Purpose of the study and specific objectives or questions | P,7 lines 133-135 |
|  |  |  |
| **Methods** | |  |
|  | **Qualitative approach and research paradigm** - Qualitative approach (e.g., ethnography, grounded theory, case study, phenomenology, narrative research) and guiding theory if appropriate; identifying the research paradigm (e.g., postpositivist, constructivist/ interpretivist) is also recommended; rationale** | P8, lines 159-161 |
|  | **Researcher characteristics and reflexivity** - Researchers’ characteristics that may influence the research, including personal attributes, qualifications/experience, relationship with participants, assumptions, and/or presuppositions; potential or actual interaction between researchers’ characteristics and the research questions, approach, methods, results, and/or transferability | P14, lines 258- 262 |
|  | **Context** - Setting/site and salient contextual factors; rationale** | P9, lines 176-192 |
|  | **Sampling strategy** - How and why research participants, documents, or events were selected; criteria for deciding when no further sampling was necessary (e.g., sampling saturation); rationale** | P9, lines 177-193 |
|  | **Ethical issues pertaining to human subjects** - Documentation of approval by an appropriate ethics review board and participant consent, or explanation for lack thereof; other confidentiality and data security issues | P13, lines 240-244  P15, lines 287 - 289 - |
|  | **Data collection methods** - Types of data collected; details of data collection procedures including (as appropriate) start and stop dates of data collection and analysis, iterative process, triangulation of sources/methods, and modification of procedures in response to evolving study findings; rationale** | P10, lines194-201 |
|  | **Data collection instruments and technologies** - Description of instruments (e.g., interview guides, questionnaires) and devices (e.g., audio recorders) used for data collection; if/how the instrument(s) changed over the course of the study | P11,lines 208-215 |
|  | **Units of study** - Number and relevant characteristics of participants, documents, or events included in the study; level of participation (could be reported in results) | P12,lines 221– 228 |
|  | **Data processing** - Methods for processing data prior to and during analysis, including transcription, data entry, data management and security, verification of data integrity, data coding, and anonymization/de-identification of excerpts | P14, lines 270-282 |
|  | **Data analysis** - Process by which inferences, themes, etc., were identified and developed, including the researchers involved in data analysis; usually references a specific paradigm or approach; rationale** | P15, lines 267-280 |
|  | **Techniques to enhance trustworthiness** - Techniques to enhance trustworthiness and credibility of data analysis (e.g., member checking, audit trail, triangulation); rationale** | P1, lines238 - 239  P13, lines 597-602 |
|  |  |  |
| **Results/findings** | |  |
|  | **Synthesis and interpretation** - Main findings (e.g., interpretations, inferences, and themes); might include development of a theory or model, or integration with prior research or theory | P16, lines 292-486 |
|  | **Links to empirical data** - Evidence (e.g., quotes, field notes, text excerpts, photographs) to substantiate analytic findings | P19, lines 330-331  P19, lines 334-335  P20, lines 354-355  P21, lines 361 -362  P21, lines366– 368  P21, lines 375-376  P22, lines 379 -382  P22, lines 394 -396  P22, lines 398 -399 |
|  |  |  |
| **Discussion** | |  |
|  | **Integration with prior work, implications, transferability, and contribution(s) to the field -** Short summary of main findings; explanation of how findings and conclusions connect to, support, elaborate on, or challenge conclusions of earlier scholarship; discussion of scope of application/generalizability; identification of unique contribution(s) to scholarship in a discipline or field | P27, lines 488-594 |
|  | **Limitations** - Trustworthiness and limitations of findings | P33, lines 603-607 |
|  |  |  |
| **Other** | |  |
|  | **Conflicts of interest** - Potential sources of influence or perceived influence on study conduct and conclusions; how these were managed | P36, lines 625-653 |
|  | **Funding** - Sources of funding and other support; role of funders in data collection, interpretation, and reporting | P35, lines 629-630 |
|  |  |  |
|  | *The authors created the SRQR by searching the literature to identify guidelines, reporting standards, and critical appraisal criteria for qualitative research; reviewing the reference lists of retrieved sources; and contacting experts to gain feedback. The SRQR aims to improve the transparency of all aspects of qualitative research by providing clear standards for reporting qualitative research. |  |
|  |  |  |
|  | **The rationale should briefly discuss the justification for choosing that theory, approach, method, or technique rather than other options available, the assumptions and limitations implicit in those choices, and how those choices influence study conclusions and transferability. As appropriate, the rationale for several items might be discussed together. |  |
|  |  |  |
|  | **Reference:** |  |
|  | O'Brien BC, Harris IB, Beckman TJ, Reed DA, Cook DA. **Standards for reporting qualitative research: a synthesis of recommendations.** *Academic Medicine*, Vol. 89, No. 9 / Sept 2014  DOI: 10.1097/ACM.0000000000000388 |  |
|  |  |  |
|  |  |  |
